# Multiple Roles of sFRP2 in Cardiac Development and Cardiovascular Disease

**DOI:** 10.7150/ijbs.40923

**Published:** 2020-01-14

**Authors:** Yu Wu, Xinyue Liu, Haoxiao Zheng, Hailan Zhu, Weiyi Mai, Xiaohui Huang, Yuli Huang

**Affiliations:** 1 Department of Cardiology, Shunde hospital, Southern Medical University, Jiazi Road 1 Lunjiao Town, Shunde District, Foshan, Guangdong, 528308, China.; 2 Department of Cardiology, The First Affiliated Hospital of Sun Yat-sen University, 510080, Guangzhou; 3 The George Institute for Global Health, NSW 2042 Australia

**Keywords:** Wnt Signaling, Secreted frizzled-related protein 2, Cardiac Fibrosis, Angiogenesis, Hypertrophy, Cardiac development

## Abstract

The Wnt signaling pathway plays important roles in organ development and disease processes. Secreted frizzled-related protein 2 (sFRP2), a vital molecule of Wnt signaling, can regulate cardiac development and cardiovascular disease. Recent studies have suggested that sFRP2 is not only an antagonist of the canonical Wnt signaling pathway, but also has a more complex relationship in myocardial fibrosis, angiogenesis, cardiac hypertrophy and cardiac regeneration. Here, we review the role of sFRP2 and Wnt signaling in cardiac development and cardiovascular disease.

## Introduction

The heart is one of the most essential organs for maintaining the normal operation of activities of daily living, and the development of the embryo heart and cardiovascular disease are complex processes. Exploring the regulatory mechanisms that underlie these processes will allow a deeper understanding of cardiac development and provide new therapeutic targets for cardiovascular disease.

The Wnt signaling pathway is important for maintaining the homeostasis of embryonic development, and participates in the regulation of biological processes that include cell proliferation, differentiation, apoptosis, and cell localization 1, 2.On one hand, during cardiac development, Wnt signaling is an important regulator of early cardiac stimulation. On the other hand, the Wnt signaling pathway is also involved in the regulation of cardiovascular diseases, such as cardiac fibrosis, myocardial infarction and cardiac hypertrophy.

The proteins of secreted frizzled-related protein (sFRP) family are antagonists of the Wnt signaling pathway, and sFRP2 is considered to be the most potent 3. Recent studies showed that the role of sFRP2 is not only an antagonist for the canonical Wnt signaling pathway, but it also has a more complex relationship with the Wnt pathway in myocardial fibrosis and cardiac regeneration. This review will focus on the importance of sFRP2 in cardiac development and cardiovascular disease.

## Overview of Wnt Pathways and sFRP2

The term “Wnt” is derived from a combination of two words: wingless (*wg*), the segment polarity gene of fruit flies, and the mouse proto-oncogene *int-1*. In 1982, Nusse et al. first discovered a *Wnt* gene in the study of a viral transcriptional mechanism in the mouse mammary gland that was found to transmit growth and development signals between cells 4. Nineteen *Wnt* genes have been found in mammals. Wnt proteins bind receptors on the cell membrane in an autocrine or paracrine manner to regulate target gene expression. The Wnt signaling pathway is widely involved in early embryo development, cell proliferation, migration, tumorigenesis, metastasis, and stem cell growth regulation 5. Currently, three major pathways for Wnt signaling have been clearly described: the canonical Wnt/β-catenin pathway, the non-canonical Wnt/c-jun pathway, and the Wnt/Ca^2+^/ PKC pathway 6.

**Figure 1:**
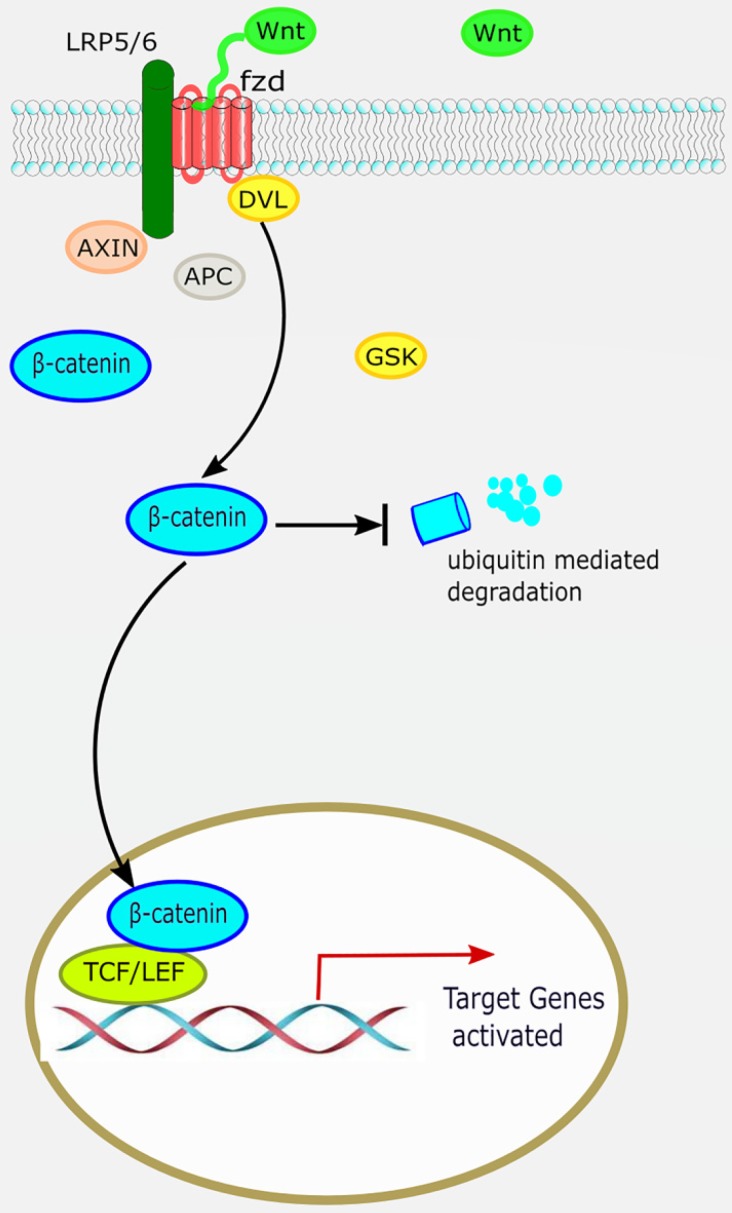
** The canonical Wnt/β-catenin pathway.** When the canonical Wnt/β-catenin pathway is “on”, the receptor complex consisting of frizzled and low-density lipoprotein receptor-related protein (LRP)5/6 bind WNT, which recruits the disheveled (DVL) protein to the plasma membrane. Subsequently, several components of the β-catenin destruction complex are recruited to the membrane, where they inhibit β-catenin ubiquitination and degradation, leading to stable accumulation of β-catenin in the cytoplasm. Lymphocyte enhancement factor/ T-cell factor (LEF/TCF) then binds to β-catenin to regulate the expression of target genes downstream of the Wnt pathway.

The canonical Wnt/β-catenin pathway is widely involved in organ development, histogenesis, regulation of the behavior and function of various precursor cells, meanwhile it is also an important regulatory factor in cardiac development. β-catenin is the most important signaling molecule of the canonical Wnt pathway, with its stability and nuclear translocation being the most important markers of canonical Wnt activation. The basic processes of the canonical Wnt/β-catenin signaling pathway are as follows: ligand Wnt protein combines with frizzled (*Fz*) receptor to form a large complex on the cell surface with low-density lipoprotein receptor-related protein (LRP)5/6 to activate scattered protein (*Dsh/Dvl*), and affect the distribution of intracellular axis protein Axin. Therefore, inhibiting the phosphorylation activity of GSK3-β leads to stable accumulation of β-catenin in the cytoplasm 7. β-catenin translocates to the nucleus and combines with lymphocyte enhancement factor/T-cell factor (LEF/TCF) to regulate the expression of target genes downstream of the Wnt pathway, such as *c-Myc* and *cyclin D1* (Figure 1) 8.

**Figure 2:**
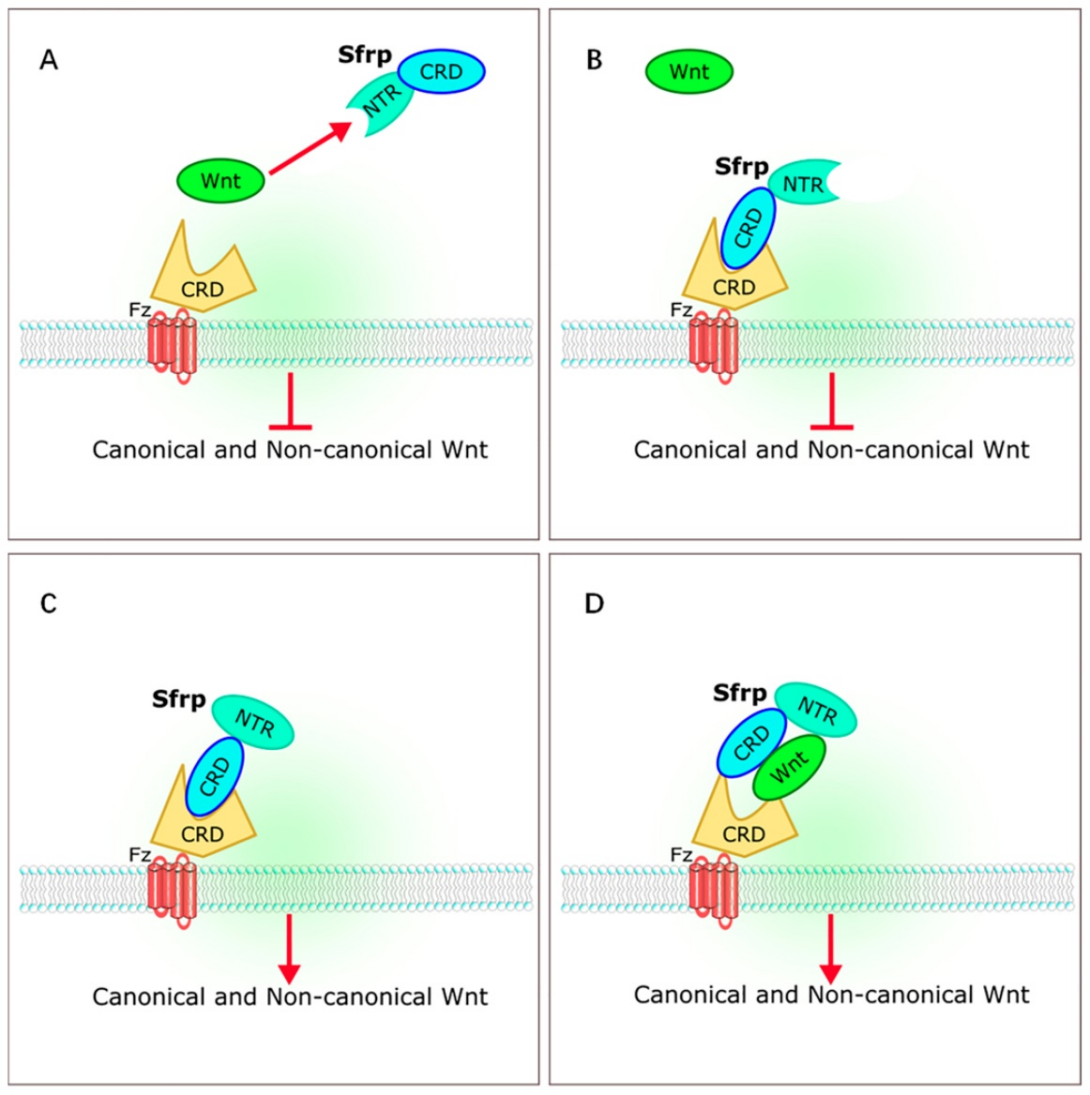
** Interaction of sFRPs and Wnt signaling pathways.** (A,B) sFRPs bind to Wnt through a cysteine-rich domain (CRD) or a C-terminal netrin-like domain (NTR), or bind to the frizzled receptor to form a non-functional complex that inhibits Wnt signaling. (C, D) sFRP interacts to form dimers, or promotes Wnt by transporting Wnt proteins to frizzled or by binding directly to frizzled during signal transduction. sFRP, secreted frizzle-related protein.

The sFRP family has long been considered a class of antagonists of the Wnt signaling pathway. Five members of sFRP family (*Sfrp1-Sfrp5*) have been identified in mammals 4-6, 9. The sFRP family is thought to be a suppressor of Wnt signaling 10-12. However, recent studies on the relationships between sFRPs and Wnt signaling have yielded different results. Bovolenta et al. revealed that different sFRP family proteins can both inhibit and promote Wnt signaling 13. The sFRPs bind to Wnt through a cysteine-rich domain (CRD) or a C-terminal netrin-like domain (NTR), or bind to the frizzled receptor to form a non-functional complex that inhibits Wnt signaling. The sFRPs can also interact with each other to form dimers, or promote Wnt by either transporting Wnt proteins to frizzled or by binding to frizzled directly during signal transduction 13 (Figure 2).

Highly conserved throughout evolution, sFRP2 is widely involved in cell proliferation, apoptosis and differentiation. Its gene is located on human chromosome 4 and is about 2 kb in length, encoding a soluble protein of 34 kDa. The highest expression level of sFRP2 that has been found is in undifferentiated precursor adipocytes in rodents and humans 7. Previously, sFRP2 was regarded as one of the strongest antagonist of Wnt signaling. It is interesting that, along with other sFRP family members, recent studies have raised questions about the nature of the interactions of sFPR2 in Wnt signaling. Yamamura et al. first reported that sFRP2 can activate the canonical Wnt signaling pathway in renal cell carcinoma 14. Other studies also reported that sFRP2 can activate the canonical Wnt signaling pathway in different cell types and disease models, including lung cancer cells 15, dermal papilla cells 16, intestinal epithelial cells 17, the vertebrate optic disc 18, and cardiac fibroblast cells 19.

## The role of sFRP2 in cardiac development and cardiovascular disease

### Role of sFRP2 in cardiac development

During embryonic development of vertebrates, the heart, as the hub of the circulatory system, is the earliest functional organ to develop. Cardiac development originates from the cardiac progenitors of the lateral plate mesoderm, which begins to develop under the trigger of the induction signal 20. The growth of the heart is extremely complicated but can be briefly summarized as four stages: 1) the establishment of the anterior plateau of the mesoderm; 2) the specialization of cardiomyocytes and endocardial progenitor cells; 3) the concentric regions of the foreguts merge to form a linear heart tube; and 4) the heart tube is cyclized and transformed into a four-chamber heart 21, 22. Liu et al. first discovered that Wnt3a deficiency leads to the decreased expression of mesoderm markers 23. Huelskenet al. created a knockout of β-catenin in mouse embryos and found that the mesoderm of the heart could not form, suggesting the cardiac development, particularly the mesoderm development, is dependent on activation of the canonical Wnt pathway 24. Subsequent studies have also shown that the activation of canonical Wnt siganling pathway is essential in various stages of cardiac development, including development of cardiac neural crest cell development and outflow tract, and coronary artery formation 25, 26.

Nevertheless, a study of chicken and Xenopus embryos suggested that the formation of the heart depends on lower Wnt/β-catenin activity 27. How might we explain these contradictory results? Naito et al. first proposed a two-way theory of Wnt/β-catenin signaling on cardiac developmental regulation. They found that during the differentiation of embryonic stem (ES) cells into cardiomyocytes, Wnt signaling promotes the differentiation of ES cells in the early stage, then plays a negative regulatory role in the late stage 28. Ueno et al. found that, if the canonical Wnt signal is activated before the formation of the primitive gut, specialization of mesoderm cells can be stimulated and converted into a linear heart tube; in the gastrula stage, the Wnt signal must be inhibited to allow the heart to continue to develop 29. These results led to the hypothesis that canonical Wnt/β-catenin signaling regulates cardiac development in a bidirectional manner.

Experiments in the mouse teratoma cell line P19CL6 suggested that sFRP2 functions are dependent on the positive feedback effect of destroying autologous *Wnt3a* transcription, which prevents mesoderm cell specialization and inhibits P19CL6 cells from differentiating into cardiomyocytes 30. Eisenberg et al. found that sFRP2 is expressed in the Spemann organizer and its lateral aspect in *Xenopus laevis*. In a study of chicken embryos, sFRP2 was not expressed in the endoderm but only in mesoderm and ectoderm derivatives 31. During subsequent cardiac development, sFRP2 is expressed in all parts of the heart, suggesting that sFRP2 may be involved in cardiomyocyte differentiation at the cellular level, and cell migration and cardiac development throughout the development of the heart 32, 33.

Overall, sFRP2 exhibits spatiotemporal-specific expression during cardiac development and has bi-directional regulatory effects on the canonical Wnt signaling pathway during cardiac development. Interestingly, some studies have shown that sFRP2 can also regulate the non-canonical Wnt pathways. In mice for example, sFRP2 regulates the formation of the trunk by regulating the Wnt/PCP pathway 34. During adult stem cell formation of Xenopus laevis intestine, sFRP2 regulates not only the canonical Wnt pathway, but also the non-canonical Wnt5a/Ror2 pathway 35.

### Role of sFRP2 in cardiac fibrosis

Wnt activity is normally low in the adult organism, and it is generally believed that it is reactivated under pathological conditions. It was reported that Wnt signaling is activated shortly after myocardial infarction and is significantly associated with cardiac remodeling and fibrosis 36-38. Duan et al. found that the canonical Wnt/β-catenin pathway was activated after acute ischemic myocardial injury in mice. Wnt1, a canonical Wnt, was upregulated by 8-fold in the heart within 48 hours, and cardiac fibroblasts were induced to proliferate and express profibrotic genes, eventually leading to heart repair and fibrosis 39. After inhibiting Wnt signaling in epicardial and cardiac fibroblasts, collagen deposition and loose granulation tissue at the heart lesion area were severely reduced, and cardiac function deteriorated significantly 39. Similarly, Lal et al. found that, in hamsters with ischemic heart cardiomyopathy, activation of the canonical Wnt pathway via inhibition of GSK3-β promoted cardiac fibrosis by upregulating the proliferation of cardiac fibroblasts 40.

It has been reported that sFRP2 can promote the proliferation of cardiac fibroblasts by activating the Wnt/β-catenin pathway 19. Kobayashi et al. first reported that sFRP2 has a function of promoting fibrosis. Their study found that the degree of myocardial fibrosis after myocardial infarction was reduced in sFRP2 knockout mice compared with normal mice 41. Mastri et al. found that the administration of sFRP2 antibodies in hamsters with cardiomyopathy decreased apoptosis of cardiomyocytes, reduced myocardial fibrosis and improved cardiac function 42. These findings support the proposal that sFRP2 can act as a profibrotic mediator 43. Kobayashi and Mastri et al. proposed the following possible mechanism for sFRP2 promoting fibrosis. Procollagen processing of tolloid-like proteases exerts a rate-limiting effect during myocardial fibrosis, whereas sFRP2 enhances procollagen C protease activity in mammalian bone morphogenetic protein 1 (BMP1), resulting in the conversion of procollagen to collagen in the extracellular matrix, ultimately promoting collagen deposition and fibrosis 41, 42. Similarly, Schuetze et al. revealed that, in cardiac fibroblasts, sFRP2 increases tissue non-specific alkaline phosphatase activity by activating the canonical Wnt signaling pathway, which promotes myocardial fibrosis and vascular calcification 44.

However, He et al. found that the degree of myocardial fibrosis was also relatively reduced in a myocardial infarction mouse model injected with recombinant sFRP2 molecules 45. In addition, other research also demonstrated that sFRP2 can play a role in reducing myocardial fibrosis via regulating the BMP1 pathway 46. Interestingly, these studies all noted that sFRP2 appears to affect cardiac fibrosis by affecting the BMP1 pathway, rather than through the canonical Wnt signaling pathway. Recently, Zhu et al. found that sFRP2 and procollagen C-proteinase enhancer 1 exert a synergistic effect on BMP1 in collagen formation. After silencing sFRP2 *in vivo*, Wnt signaling was inhibited, and collagen deposition and fibrosis were reduced 47.

**Figure 3:**
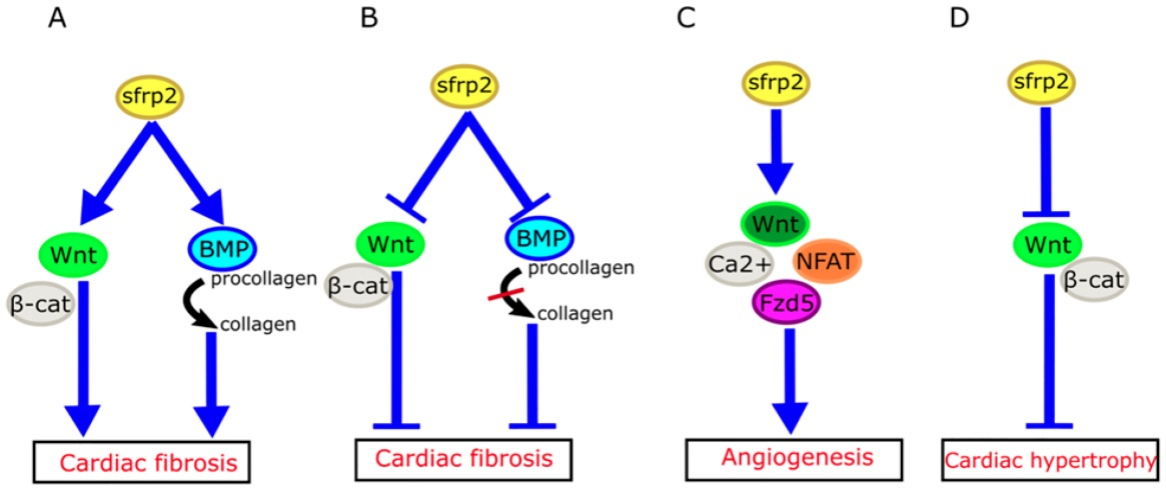
** Role of sFRP2 in cardiovascular disease.** (A) sFRP2 at low concentration activates the canonical Wnt/β-catenin pathway and enhances procollagen C protease activity in mammalian bone morphogenetic protein 1 (BMP1), eventually lead to cardiac fibrosis. (B) sFRP2 at high concentration inhibits the canonical Wnt/β-catenin pathway and attenuates procollagen C protease activity in mammalian BMP1, eventually leading to inhibition of cardiac fibrosis. (C) sFRP2 induces endothelial angiogenesis via the non-canonical Wnt/Ca^2+^/NFAT/Fzd5 pathway. (D) sFRP2 protects the heart from hypertrophy by inhibiting the canonical Wnt/β-catenin pathway. sFRP, secreted frizzle-related protein.

How might we explain the apparently inconsistent role of sFRP2 in myocardial fibrosis in these studies? Mastri et al. speculated that high doses of sFRP2 can effectively inhibit the canonical Wnt signaling in the myocardium, while producing anti-fibrotic effects 42. Similarly, the work of Alfaro et al. has revealed that low levels of sFRP2 (<1 µg/mL) promote procollagen C protease activity, which in turn promotes fibrosis, whereas high concentrations of sFRP2 (>6 µg/mL) has the opposite effect 48. Based on these studies, we propose that sFRP2 may play a similar two-way role in myocardial fibrosis (Figure 3A, B). A low concentration of sFRP2 can enhance the effects of Wnt pathway and promote myocardial fibrosis, while a high concentration of sFRP2 can antagonize the Wnt pathway and inhibit myocardial fibrosis. This bidirectional effect appears to be quite common during the fibrotic process in other organs. For example, Rajasekaran et al. observed that high-dose sFRP2 injection can inhibit Wnt signaling and reduce fibrosis in anal external sphincter injury in rabbits 49.

We do not yet completely understand the specific mechanisms of sFRP2 in fibrosis. Genetic differences in model systems, crosstalk between Wnt and other signaling pathways, and the bidirectional effects of sFRP2 on the Wnt signaling pathway highlight the complexity of the relationship between sFRP2 and cardiac fibrosis. It is possible that sFRP2 plays different roles at different stages of the development of myocardial fibrosis.

### Role of sFRP2 in angiogenesis

Angiogenesis is a complex process which can be summarized as four steps: the induction of tip cells, sprout elongation, vascular branching and stabilization of the newly formed vascular network 50. A large number of studies have proved that Wnt proteins play an important role in regulating the function of endothelial cells, tip cells, stalk cells during angiogensis via either canonical Wnt or non-caonical Wnt signaling pathway 51-53.

sFRP2 exerts pro-angiogenic effects through activation of non-canonical Wnt/Ca^2+^ pathways. In mouse and chicken chorioallantoic membranes, sFRP2 was also found to induce angiogenesis 54. It inhibits hypoxia-induced endothelial cell apoptosis, and induces endothelial angiogenesis by increasing endothelial cell migration. However, sFRP2 achieves the above functions through the Wnt/Ca^2+^/NFAT (nuclear factor of activated T cells) pathway, without affecting the canonical Wnt pathway 55. Similarly, sFRP2 has been shown to enhance angiogenesis in breast cancer through the above pathway 56. A recent study revealed that frizzled-5 (*Fzd5*) also plays an important role during angiogenesis promoted by sFRP2. Endothelial cell tubes fail to form when *Fzd5* is silenced in endothelial cells 57.

It is also generally understood that sFRP2 can exert an angiogenic effect through the above pathways in a wide variety of human tumors, including angiosarcoma, prostate cancer, renal cell carcinoma, lung cancer, and pancreatic cancer 58-60. Crowley and coworkers reported that the addition of exogenous sFRP2 to adipose tissue promoted the expression of vascular endothelial growth factor mRNA, which suggested that sFRP2 may have a proangiogenic function in adipose tissue (Figure 3C) 61.

### Role of sFRP2 in cardiac hypertrophy

Cardiac hypertrophy is an adaptive response of heart under many pathological states such as pressure overload and β-adrenergic stimuli 62. Different signaling pathways and key molecules were involved in this process, including NFAT, phosphoinositide-3 kinase/protein kinase B (PI3K/AKT), extracellular regulated protein kinases (ERK), c-Jun N-terminal kinase (JNK), calcineurin and Wnt signaling pathways 62, 63. He et al. reported that Wnt3a and Wnt5a were increased in hypertrophy mouse model induced by isoprenaline, which indicated that both canonical and non-canonical Wnt were involved in cardiac hypertrophy 64.

The HyperGEN study used linkage analysis in siblings with hypertension to determine that genetic variation in sFRP2 was associated with left ventricular hypertrophy 65. Furthermore, a recent study has shown that sFRP2 can exert anti-atrophic effects on muscle cells by inhibiting transforming growth factor-β1 in muscle cells 66. Mohamed et al. have found that plasma membrane calcium ATPase (PMCA)4 acts as a key regulator of pathological cardiac hypertrophy by regulating sFRP2. Pharmacological blocking of PMCA4 can increase the expression of sFRP2 in cardiac fibroblasts, downregulate the canonical Wnt signaling pathway, and protect cardiomyocytes from pathological hypertrophy. These results suggest that sFRP2 may play a protective role in myocardial pathological hypertrophy 67 (Figure 3D).

### Role of sFRP2 in cardiac regeneration therapy

The limited regenerative potential of cardiomyocytes has made cardiac regeneration therapy by stem cell transplantation a hotspot in cardiovascular disease research 68. The therapeutic effect of stem cell transplantation depends on the survival and transplantation ability of these cells in the target organs. Due to the influence of local myocardial inflammation and fibrosis, the survival rate of stem cells after transplantation is low, significantly affecting the efficiency of cardiac transplantation 69. To improve the effect of cell therapy, it will be necessary to enhance the viability of stem cells in the inflammatory and fibrotic environment created by ischemia-reperfusion injury.

Mesenchymal stem cells (MSCs) are believed to be a promising candidate stem cell for cardiac therapy 70, 71. Under hypoxic conditions, sFRP2 is thought to be a key regulator of the PI3K/AKT pathway in MSCs 48. Blockage of the PI3K/Akt pathway or knockdown of sFRP2 with siRNA significantly increases the apoptosis of MSCs 72. Gehmert et al. found that insulin-like growth factor-1 (IGF-1) plays an anti-apoptotic role by mediating sFRP2 activation in adipose tissue-derived stem cells; the release of sFRP2 is also dependent on the PI3K/Akt pathway 73. Mirotsou et al. suggested that overexpression of Akt in MSCs can significantly increase their viability and survival rate after transplantation into the infarct myocardium, reduce the size of myocardial infarction, increase neovascular density, and ultimately improve cardiac function. These effects were dependent on the expression of sFRP2 as knocking out *Sfrp2* can abolish the aforementioned benefits 74. After the injection of MSCs overexpressing sFRP2 around the infarcted myocardium, Alfaro et al. observed a downregulation of the canonical Wnt pathway 75. Compared with the control group, the number of surviving MSCs was increased after myocardial injury, accompanied by increased vascular density, decreased infarct size, and significant improvement in cardiac function 75. Pomduk et al. found that sFRP2 improved the viability of human MSCs (hMSCs) under oxidative stress, thereby improving the therapeutic effect of stem cell transplantation. However, sFRP2 did not affect the cell morphology, surface marker expression or differentiation potential of hMSCs 76. This accumulation of evidence demonstrated that activation of the PI3K/Akt/sFRP2 pathway can protect the myocardium from apoptosis after MSCs transplantation 77, 78. Our group also found that IGF-1 can promote the proliferation of MSCs and expression of C-X-C chemokine receptor type 4 (CXCR4), further promoting the migration of MSCs 79. Moreover, we found that MSCs transplanted into rats with myocardial infarction can promote the expression of the anti-apoptotic gene *Bcl-2*, inhibit cardiomyocyte apoptosis, and improve cardiac function 80.

Apart from MSCs, sFRP2 also has multiple effects on the properties of other types of stem cells. Aubert et al. found that sFRP2 can promote the differentiation of embryonic stem cells into neural progenitors by antagonizing the canonical Wnt signaling pathway 81, and subsequent studies have found that sFRP2 plays a key role in adipogenic and neuronal differentiation of dental tissue-derived MSCs 82. Schmeckpeper et al. found that sFRP2 can enhance the differentiation of cardiac progenitor cells (CPCs) *in vivo* 83. After myocardial ischemia-reperfusion injury, sFRP2 can double the number of new cardiomyocytes differentiated from CPCs, reduce infarct size, and improve cardiac function significantly. In this process, the canonical Wnt/β-catenin signaling pathway was inhibited by sFRP2. At the same time, sFRP2 induces CPC cell cycle arrest and upregulates cardiac transcription factors by activating the non-canonical Wnt/JNK and Wnt/CaMKII pathways, which induce differentiation of CPCs into cardiomyocytes 83.

## Summary and future perspectives

SFRP2 plays an important role in the process of heart development and a various cardiovascular pathophysiological condition. In myocardial fibrosis, sFRP2 may play a bidirectional effect depending on the different stage of disease and concentration. Subsequent studies had shown that sFRP2 can promote angiogenesis by activating the non-canonical Wnt/Ca^2+^/NFAT pathway, while without affecting the canonical Wnt pathway. Furthermore, sFRP2 has been shown to downregulate the canonical Wnt signaling pathway, and protect cardiomyocytes from pathological hypertrophy. In cardiac regeneration therapy, sFRP2 can improve the survival of stem cells in the adverse environment, and enhance the therapeutic effects for myocardial infarction. Therefore, we believe that sFRP2 is a promising therapeutic target for multiple pathophysiological conditions of cardiovascular disease. However, the specific mechanisms of sFRP2 in these pathophysiological processes require further exploration. Interventions in the Wnt signaling pathway using modified sFRP2 expression may be a promising area of research for heart diseases.

## Abbreviations

BMP1: bone morphogenetic protein 1
CPCs: cardiac progenitor cells
CRD: cysteine-rich domain
CXCR4: C-X-C chemokine receptor type 4
ERK: extracellular regulated protein kinases
ES: embryonic stem
Fz: frizzled
IGF-1: insulin-like growth factor-1
JNK: c-Jun N-terminal kinase
LEF/TCF: lymphocyte enhancement factor/T-cell factor
LRP: low-density lipoprotein receptor-related protein
MI: myocradial infarction
MSCs: mesenchymal stem cells
NFAT: nuclear factor of activated T cells
NTR: C-terminal netrin-like domain
PI3K/Akt: phosphoinositide-3 kinase/protein kinase B
PMCA: plasma membrane calcium ATPase
sFRP2: Secreted frizzled-related protein 2
wg: wingless
